# Cannabinoid Receptor Type 1 Availability in Individuals with a History of Childhood Trauma: A Positron Emission Tomography Study

**DOI:** 10.21203/rs.3.rs-6536815/v1

**Published:** 2025-05-30

**Authors:** ANAHITA BASSIR NIA, Ardavan Mohammad Aghaei, Brian Pittman, Nachshon Korem, Deepak D’Souza, Marc Potenza, Ansel Hillmer, Mohini Ranganathan, Ilan Harpaz-Rotem

**Affiliations:** Yale University School of Medicine; Yale School of Medicine; Yaly university; Yale University School of Medicine; Yale university; Yale University; Yale University; Yale University

## Abstract

Early life adversity has a lasting impact on the endocannabinoid (eCB) system based on animal models. However, the impact of early life adversity such as childhood trauma (CT) on the eCB system has not been thoroughly studied. We assessed the availability of cannabinoid receptor type 1 (CB1R) in individuals with CT compared to healthy controls without CT (HCs). Cannabinoid receptor type 1 (CB1R) availability was compared in adults with CT (N = 22) and age- and sex-matched HCs (n = 22), using positron emission tomography (PET) imaging with the CB1R-specific radiotracer [^11^C]OMAR. Using linear models, the effect of the group was assessed on global and trauma-relevant brain regions (amygdala, hippocampus, and frontal cortex). Compared to HCs, lower CB1R availability was observed in CT globally (difference= −11.36%, *F*_*(1,42)*_ = 4.35, p = 0.04), in amygdala (−13.70%, *F*_*(1,84)*_ = 6.66, p = 0.01), and in hippocampus (−14.50%, *F*_*(1,84)*_ = 6.59, p = 0.01), but not in frontal cortex (−8.08%, *F*_*(1,84)*_ = 2.17, p = 0.14). There were no effects of a diagnosis of posttraumatic stress disorder, major depressive disorder, nicotine dependence, or the use of antidepressant medication. This preliminary result of lower CB1R availability in adults with CT compared with HCs suggests eCB dysregulation associated with CT. Future studies should replicate and extend this finding and examine the potential effects of various trauma features on the eCB system.

## Introduction

Childhood trauma (CT), defined as exposure to significant traumatic events before the age of 18, has profound impacts on mental health [1]. Individuals who experience CT are at higher risk of developing several psychiatric disorders, including depression and post-traumatic stress disorder (PTSD) [2, 3]. CT exposure has been reported to lead to structural and functional alterations in the nervous system, resulting in long-lasting consequences [4–7]. CT typically imposes threats to survival, body integrity, or sense of self, which explains the significant alterations in the ‘threat-detection and response circuit’, particularly the hippocampus, amygdala, and prefrontal cortex (PFC), in individuals with a history of CT [7].

The endocannabinoid (eCB) system, which has a central role in stress response [8], is impacted by various forms of trauma and chronic stress [9–13]. Trauma-induced reductions in cannabinoid receptor type 1 (CB1R), the most abundant G-protein coupled receptor in the brain, have been reported in animal models of early life adversity, such as maternal deprivation and social isolation [14, 15]; these findings persist into adulthood [16]. Similarly, animal models of chronic stress have reported lower levels of CB1Rs in various brain regions, including the hippocampus [9–13].

Despite this evidence from animal studies, the exact impact of CT on the eCB system in humans remains unclear. Human studies on the impact of trauma on the eCB system have predominantly focused on peripheral eCB levels and reported lower peripheral levels of 2-arachidonoyl glycerol (2-AG) and Anandamide (AEA) in those with trauma-related psychiatric disorders [17–19]. However, they often did not specifically address CT. In contrast, a few studies that investigated peripheral eCBs in individuals with CT reported higher hair levels of 1-arachidonoylglycerol (1-AG) [20], and higher plasma levels of an eCB-related compound, oleoylethanolamide (OEA) [21]. While these studies provide valuable evidence on the unique long-lasting impact of CT on the eCB system, peripheral eCB levels can originate from various sources [22], and are not correlated with central eCBs [23]. The impact of CT on the central eCB system in humans has not been reported.

CB1Rs are principal components of the eCB system that can be measured *in vivo* using PET imaging and the CB1R specific radiotracers, such as [11C]OMAR [24–27]. In this study, for the first time, we aimed to compare CB1R availability in adult humans with and without CT histories. Consistent with evidence from animal models [14, 15], we hypothesized to observe a lower CB1R availability in individuals with a history of CT versus healthy controls with no history of CT (HC), both at a whole-brain level, and in three specific brain regions that express high levels of CB1R and are largely affected by CT, i.e., amygdala, hippocampus, and frontal cortex [28].

## Methods

### Participants:

Adult men and women (age ≥ 18) with CT histories (n=22) comprised a CT group, and age- and sex-matched healthy individuals without CT histories served as the HC group (n=22). All individuals received detailed information about the study and signed written informed consent. Inclusion in the CT group was determined based on exposure to a major traumatic event based on Criterion A of the DSM-5 criteria for PTSD, assessed by the Clinician-Administered PTSD Scale for DSM-5 (CAPS-5) or Structured Clinical Interview for DSM-5 Disorders (SCID-5), and exposure to traumatic events for the first time before the age of 18, assessed by the Trauma Questionnaire (TQ) and Childhood Trauma Questionnaire (CTQ) [29–32]. Nicotine dependence was assessed using the Fagerstrom Test for Nicotine Dependence (FTND) [33]. Exclusion criteria included severe mental illness (e.g., schizophrenia), current substance use disorder (excluding nicotine), recent cannabis use, use of anticoagulants, and any medical condition that could interfere with the study, as determined by the primary investigator. The study protocol was approved by the institutional review boards of Yale School of Medicine and the VA Connecticut Health Care System. Additional HCs were included from our group’s previously published research investigating CB1R availability in individuals with other psychiatric disorders [34, 35].

### Radiochemistry and Imaging:

Eligible participants underwent structural Magnetic Resonance Imaging (MRI) using a Siemens 3T system (Siemens Medical Solutions, Malvern, Pennsylvania). Standard T1-weighted anatomical sequences were obtained to exclude anatomical abnormalities and to co-register with PET scan images. [^11^C]OMAR, a specific CB1R tracer, was used to measure CB1R availability. [^11^C]OMAR was synthesized with high molar activity according to a previously published protocol adapted to the TRACERlab FXC Pro automated synthesis module (GE Healthcare, Milwaukee, WI) [36]. Dynamic PET scans were acquired using a High Resolution Research Tomograph (HRRT, Siemens Medical Systems, Knoxville, TN) in 3D mode. Imaging started with a six-minute transmission scan used for attenuation correction. Subsequently, participants were scanned for 120 minutes following an intravenous bolus injection of 12.8 (4.16) mCi [^11^C]OMAR over a period of one minute. To account for motion, an optical system (Polaris Vicra, Northern Digital Incorporated, Waterloo, Ontario, Canada) was positioned behind the PET scanner to track the position of an infrared reflective tool mounted on the participant’s head. An arterial line was placed prior to the scan with arterial blood sampling acquired throughout the imaging session. Blood samples collected at 5, 15, 30, 60, 90, and 120 minutes post-injection were analyzed using column-switching high-performance liquid chromatography (HPLC) to measure the unmetabolized parent tracer fraction for calculation of the metabolite-corrected arterial plasma input function [37, 38].

### Image Processing:

Consistent with previous work, dynamic list mode data were reconstructed and corrected for attenuation, normalization, scatter, randoms, dead time, and motion using the ordered subset-expectation maximization (OSEM) algorithm [39]. Subsequently, PET images were registered to the participant’s MR anatomical image and then to an MR template image [40]. Anatomical Automatic Labeling (AAL) was used to define thirteen regions of interest (ROIs), namely amygdala, caudate, cerebellum, anterior cingulate cortex, posterior cingulate cortex, frontal, hippocampus, insula, occipital, parietal, putamen, temporal, and thalamus [41]. Partial volume correction was also applied using a method similar to previous studies [40, 42]. Time-activity curves for each ROI were extracted, and multilinear analysis-1 (t* = 30 min) was used to estimate [^11^C]OMAR *V*_*T*_ in each region [43].

### Statistical Analysis:

All outcomes were assessed for normality using normal probability plots and Kolmogorov-Smirnov tests. Similar to previous studies [19, 34, 44], a global composite of CB1R availability was calculated by averaging [^11^C]OMAR *V*_*T*_ across all 13 study regions and served as a primary outcome and was compared between groups (CT vs HC) using a linear model. Additionally, three ROIs (amygdala, frontal cortex, and hippocampus) were selected as primary outcomes, given the evidence on the heavy impact of CT on these regions [7]. These were analyzed using a linear mixed model (LMM) with group as a between-subjects factor and region as a within-subjects factor. The interaction was modeled, and the correlation between observations measured repeatedly within each subject was modeled using structured variance-covariance matrices. The best-fitting model was selected according to information criteria, and residual plots confirmed model fit. Least-square (LS) means were estimated and compared post-hoc. The rest of the ten ROIs were considered as exploratory outcomes and were analyzed using similar LMMs. Previous studies have suggested that age, sex, and body mass index (BMI) relate to CB1R availability in some ROIs [40]; therefore, these were assessed in each model. However, their inclusion did not alter the model results and had no significant effect on the outcomes, and thus, they were dropped from the model for parsimony. Potential effects of clinical factors within CT (current psychiatric diagnosis, medication use, number of trauma events, nicotine dependence) on global CB1R were assessed using similar linear models as described above. All primary outcomes were tested using a two-sided alpha=0.05 significance threshold. For exploratory analyses testing for group differences in secondary regions and for the effects of clinical factors within the CT group, Type I error was corrected using the less conservative step-down (SD) Sidak adjustment [45]. All analyses were conducted using SAS, version 9.4 (SAS Institute Inc., Cary, NC, USA).

## Results

### Baseline characteristics.

Age, sex, BMI, and the dose and mass of the tracer injected during the PET imaging were similar between groups (Table 1). Of the 22 participants with CT, 8 met criteria for current posttraumatic stress disorder (PTSD), and 7 met criteria for current major depressive disorder (MDD, 6 of whom had both MDD and PTSD) (based on SCID-5). Six individuals with CT were taking antidepressant medications.

### CB1R availability in the CT versus HC groups.

**Figure 1:**
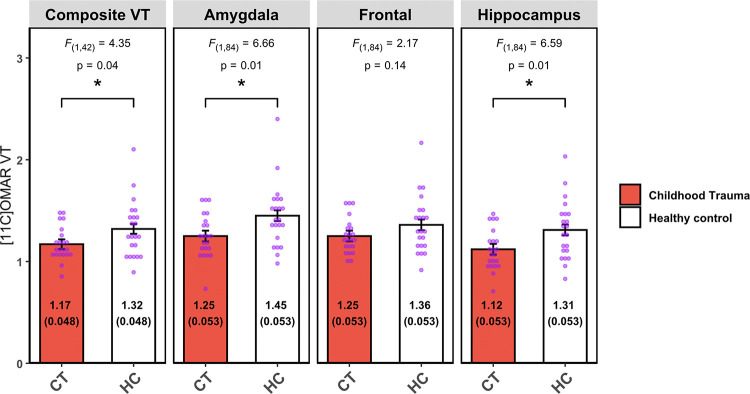
[^11^C]OMAR volume of distribution LS mean (standard error) as a global composite value and in the three PTSD-related ROIs.

Lower composite CB1R availability was observed in the CT versus HC groups (difference −11.36%, F_(1,42)_=4.35; p = 0.04, d = 0.63). In the LMM examining the pre-selected 3 PTSD-relevant ROIs, the group-by-region interactions was significant (*F*_*(2,84)*_=4.49, p=0.014) driving by lower CB1R availability among CT participants in the amygdala (−13.70%, *F*_*(1,84)*_=6.66, p=0.01, d=0.72) and hippocampus (−14.50%, *F*_*(1,84)*_=6.59, p=0.01, d=0.78), but not in the frontal region (−8.08%, *F*_*(1,84)*_=2.17, p=0.14, d=0.47) (Figure1). In the exploratory analysis of other ROIs, no statistically significant differences were observed (Table 2).

### The effect of clinical factors on global CB1R availability among CT

There was no difference in CB1R availability based on meeting criteria for PTSD (*F*_*(1,20)*_=4.01, p_SD Sidak_=0.27) or MDD (*F*_*(1,20)*_=4.11, p_SD Sidak_=0.27). On average, CT participants reported encountering 19.0 ± 33.4 traumatic events in their lifetime, and event frequency was not associated with CB1R availability (Spearman r = −0.05, p_SD Sidak_ = 0.84). No differences were observed between participants who were or were not taking antidepressant medications (*F*_*(1,20)*_=2.63, p_SD Sidak_=0.32). While there was no significant difference in CB1R availability between tobacco users and non-users (*F*_*(1,20)*_=0.51, p_SD Sidak_=0.71), this result should be interpreted with caution, as only two CT participants reported smoking.

## Discussion

Whole-brain CB1R availability is lower in adults with a history of CT compared to controls with a reduction in specific regions, including the hippocampus and amygdala. To the best of our knowledge, this is the first report on the *in vivo* CB1R availability in adult humans with CT. Our main finding aligns with most preclinical studies that have reported lower CB1R immunoreactivity, expression levels, or binding following early life stressors in various brain regions, especially the hippocampus, with findings persisting into adulthood [14, 46–48]. However, not all preclinical studies have reported such findings [16, 49].

Both animal and human studies have demonstrated that CB1R expression gradually increases during early childhood, reaches its maximum in adolescence, and then drops to stable levels in early adulthood [50]. Thus, exposure to CT, when the eCB system is still immature, could disrupt the normal development of the main elements of the eCB system, such as CB1R. In addition, the eCB system and the hypothalamic-pituitary-adrenal (HPA) axis interact closely in the initiation and termination of stress responses [51]. CT has been shown to dysregulate the coupling of the glucocorticoid and eCB systems, leading to impaired regulation of stress responses by glucocorticoids [52]. Chronic stress typically induces downregulation of presynaptic CB1Rs, which is likely due to the activation of genomic glucocorticoid receptors and could be blocked by glucocorticoid receptor antagonists [12, 13]. While the precise mechanisms for glucocorticoid receptor activation leading to downregulation of CB1Rs are not fully understood, two mechanisms have been proposed [8]: 1) direct negative regulation of the CB1R gene by glucocorticoids [53], or 2) glucocorticoid-mediated recruitment of eCB signaling, resulting in agonist-induced receptor desensitization, as observed with sustained 2-AG signaling elevation [54].

Considerable research has focused on the potential role of epigenetic programming in the delayed effects of CT on the eCB system. These studies generally indicate that CT has a persistent impact on gene expression and behavior through epigenetic mechanisms, with the HPA axis and glucocorticoid receptors being among the most consistently affected systems. A systematic review reported that CT, both in animal models and human studies, results in increased methylation and lower expression of glucocorticoid receptors [55]. This implies glucocorticoid resistance and dysregulation of the HPA axis response to stress in adulthood [55, 56]. Other studies indicate that CT impairs the coupling of the glucocorticoid and eCB systems in the hippocampus, resulting in impaired stress regulation later in life [52]. Moreover, genetic studies have found significant interactions between CT, particularly sexual abuse, and eCB-related gene polymorphisms in amygdala-related stress habituation and symptoms of cannabis use disorder [57, 58]. Our findings support the potential specific impact of CT on the eCB system, which may differ from the effects of trauma exposure when it occurs during adulthood.

Future directions: Further studies are required to replicate these findings and investigate the clinical implications of low CB1R availability in individuals with a history of CT. The availability of cannabinoids and the recent development of eCB modulators make it possible now to target the eCB system in recovery from CT [59]. Moreover, given the critical development of the eCB system in adolescence, future studies should investigate the differential impact of trauma exposure during early childhood versus adolescence. The effects of other factors, such as the cumulative effects of major traumatic events and chronic stress, or the types of trauma, are also important to investigate.

### Limitations

Study limitations warrant consideration. First, the sample size was small, but within the range of other published exploratory PET imaging studies on the eCB system [19, 34, 44]. Nevertheless, these preliminary findings warrant further research with larger sample sizes and more granular information on CT to investigate the impact of various trauma-related factors. Second, the small sample size did not allow for the investigation of sex differences. Sex differences in the eCB system and stress responses have been reported [60]. Future research should address potential sex differences in CB1R availability among individuals with CT histories. Third, not all HC participants completed questionnaires related to childhood trauma, and the extent of childhood trauma was not fully assessed in all cases. However, any history of CT in a subset of the HCs would likely attenuate rather than inflate the main findings. Lastly, we did not investigate the clinical impact of lower CB1R availability in individuals with CT histories. Further studies are needed to examine how low CB1R availability in individuals with CT histories may relate to stress responses or clinical symptoms.

## Conclusions

This exploratory study showed lower CB1R availability in individuals with a history of CT, raising the intriguing possibility of targeting the eCB in the treatment of CT consequences on mental health.

## Figures and Tables

**Table 1:** The baseline characteristics of participants.

	CT (N=22)	HC (N=22)	*p* vlaue^1^
**# of female participants; n(%)**	4 (18.2%)	4 (18.2%)	1.00
**Age; mean(SD)**	42.5 (10.1)	39.4 (7.93)	0.251
**BMI; mean(SD)**	29.8 (6.22)	27.6 (5.14)	0.214
**Tracer dose; mCi; mean(SD)**	12.8 (4.16)	13.8 (4.00)	0.553
**Tracer mass; μg/kg; mean(SD)**	0.03 (0.02)	0.03 (0.02)	0.682
**Current PTSD; n (%)**	8 (36.4%)	-	
**# of traumatic events; n(%)**	19.0 (33.4)	-	
**Nicotine dependence; n (%)**	2 (9.09%)	-	-
**FTND Score^2^; mean (SD)**	4.5 (0.5)	-	-
**Current MDD; n(%)**	7 (38.9%)		
**Current antidepressant use; n (%)**	6 (27.3%)	-	-
**Current benzodiazepine use; n (%)**	1 (4.5%)	-	-

Abbreviations: BMI, Body Mass Index; FTND, Fagerstrom Test for Nicotine Dependence; MDD, Major Depressive Disorder; PTSD, Post-Traumatic Stress Disorder.

1 T test (continuous) and Chi-square test (categorical).

2 In those with nicotine dependence.

**Table 2:** Estimated LS Means of [^11^C]OMAR VT and LMM-Derived Statistics for Secondary Regions.

	LS mean (SE)		Contrast %	*F*	*P_SD Sidak_*
	CT	HC			
**Caudate**	0.94 (0.047)	1.11 (0.047)	−15.3%	F_(1,42)_ = 6.48	0.14
**ACC**	1.32 (0.053)	1.49 (0.053)	−11.4%	F_(1,42)_ = 5.02	0.22
**PCC**	0.99 (0.050)	1.10 (0.050)	−10.0%	F_(1,42)_ = 2.15	0.32
**Cerebellum**	1.16 (0.045)	1.27 (0.045)	−8.6%	F_(1,42)_ = 3.41	0.32
**Insula**	1.30 (0.056)	1.46 (0.056)	−10.9%	F_(1,42)_ = 4.04	0.31
**Occipital**	1.17 (0.050)	1.30 (0.050)	−10.0%	F_(1,42)_ = 3.49	0.32
**Parietal**	1.21 (0.052)	1.30 (0.052)	−6.9%	F_(1,42)_ = 1.69	0.32
**Putamen**	1.34 (0.056)	1.53 (0.056)	−12.4%	F_(1,42)_ = 5.55	0.19
**Temporal**	1.25 (0.051)	1.38 (0.051)	−9.4%	F_(1,42)_ = 3.68	0.32
**Thalamus**	0.94 (0.040)	1.04 (0.040)	−9.6%	F_(1,42)_ = 2.84	0.32

Abbreviations: ACC, Anterior Cingulate Cortex; PCC; Posterior Cingulate Cortex
